# Supplementary material for: Characteristic Evaluation of Gel Formulation Containing Niosomes of Melatonin or Its Derivative and Mucoadhesive Properties Using ATR-FTIR Spectroscopy
Source: Polymers (Basel). 2021 Apr 2;13(7):1142. doi: 10.3390/polym13071142 (PMC8038236; doi:10.3390/polym13071142)
Supplement: Supplementary file 1 [file polymers-13-01142-s001.pdf]

polymers

MDPI

Supporting Information

Characteristic Evaluation of Gel Formulation Containing Niosomes of Melatonin or its Derivative and Mucoadhesive Properties Using ATR-FTIR Spectroscopy

Prangtip Uthaiwat [1], Aroonsri Priprem [2,3], Ploenthip Puthongking [3,4], Jureerut Daduang [5], Chatchanok Nukulkit [6], Sirinart Chio-Srichan [7], Patcharee Boonsiri [8] and Suthasinee Thapphasaraphong [3,4,*]

1 Graduate School, Khon Kaen University, Khon Kaen 40002, Thailand; phangtip_11@hotmail.com
2 Faculty of Pharmacy, Mahasarakham University, Maha Sarakham 44150, Thailand; aroonsri@kku.ac.th
3 Melatonin Research Group, Khon Kaen University, Khon Kaen 40002, Thailand; pploenthip@kku.ac.th
4 Department of Pharmaceutical Chemistry, Faculty of Pharmaceutical Sciences, Khon Kaen University, Khon Kaen 40002 Thailand
5 Centre for Research and Development of Medical Diagnostic Laboratories, Faculty of Associated Medical Sciences, Khon Kaen University, Khon Kaen 40002 Thailand; jurpoo@kku.ac.th
6 Department of Thai Traditional Medicine, Rajamangala University of Technology Isan Sakon Nakhon Campus, Sakon Nakhon, 47160 Thailand; chatnuk@gmail.com
7 Synchrotron Light Research Institute (Public Organization), Nakhon Ratchasima 30000 Thailand; sirinart@slri.or.th
8 Department of Biochemistry, Faculty of Medicine, Khon Kaen University, Khon Kaen 40002, Thailand; patcha_b@kku.ac.th
* Correspondence: sutpit1@kku.ac.th; Tel.: +66869218334

Citation: Uthaiwat, P.; Priprem, A.; Puthongking, P.; Daduang, J.; Nukulkit, C.; Chio-Srichan, S.; Boonsiri, P.; Thapphasaraphong, S. Characteristic Evaluation of Gel Formulation Containing Niosomes of Melatonin or its Derivative and Mucoadhesive Properties Using ATR-FTIR Spectroscopy. *Polymers* **2021**, *13*, 1142. https://doi.org/10.3390/polym13071142

Academic Editor: Alejandro Sosnik
Received: 9 March 2021
Accepted: 29 March 2021
Published: date

Publisher's Note: MDPI stays neutral with regard to jurisdictional claims in published maps and institutional affiliations.

Copyright: © 2021 by the authors. Submitted for possible open access publication under the terms and conditions of the Creative Commons Attribution (CC BY) license (http://creativecommons.org/licenses/by/4.0/).

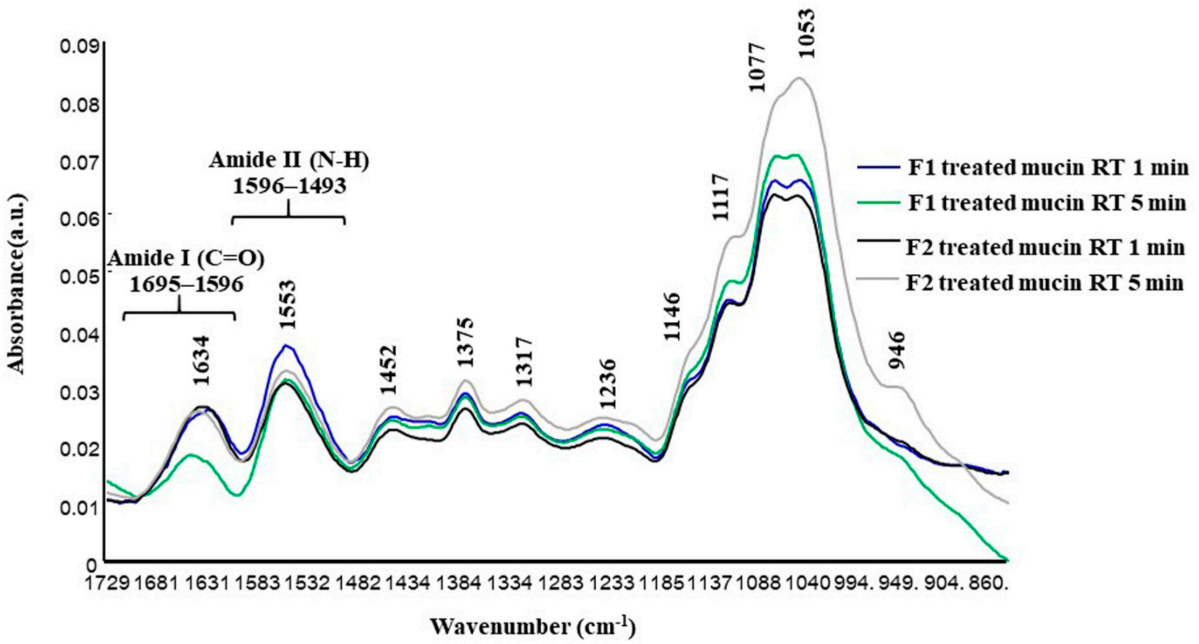

Figure S1. ATR-FTIR spectra of F1- and F2-treated mucin at RT for 1 min and 5 min, 1634 cm^{-1} (C=O stretching of amide I), 1553 cm^{-1} (C–N stretching of amide II), 1452 cm^{-1} (C–H bending), 1375 cm^{-1} (CH_3 bending), 1317 cm^{-1} and 1236 cm^{-1} (amide III), 1146 and 1117 cm^{-1} (C–O stretching), 1077 cm^{-1} (C–N stretching), 1053 cm^{-1} (C–O stretching or C+–O bending of carbohydrates), and 946 cm^{-1} (C–H out-of-plane bending). Each spectrum was averaged from 3 spectra.

Table S1. The absorption intensities under the spectra of amide I, amide II and carbohydrate, ratio of amide I/amide II and ratio of carbohydrate/amide II from F1- and F2-treated mucin at RT for 1 min and 5 min.

Peak area (raw data)	Peak1	Peak2	Peak3	Ratio	Average	Ratio	Average
Samples/Wave number(cm-1)	Amide I	Amide II	Carbo	AmideI/amideII	AmideI/amideII	Carbo/AmideII	Carbo/AmideII
	1695–1596.2	1596.2–1492.6	1189.1–972.7				
F1 treated mucin RT 1 min-1	1.240	1.822	9.214	0.681		5.057	
F1 treated mucin RT 1 min-2	1.129	1.832	8.951	0.616	0.588	4.886	5.024
F1 treated mucin RT 1 min-3	0.961	2.062	10.578	0.466		5.130	
F1 treated mucin RT 5 min-1	0.804	1.653	10.093	0.486		6.106	
F1 treated mucin RT 5 min-2	0.783	1.858	9.816	0.421	0.349	5.283	5.838
F1 treated mucin RT 5 min-3	0.181	1.309	8.019	0.138		6.126	
F2 treated mucin RT 1 min-1	1.541	1.814	10.846	0.850		5.979	
F2 treated mucin RT 1 min-2	1.368	1.469	11.577	0.931	0.878	7.881	6.613
F2 treated mucin RT 1 min-3	1.576	1.846	11.035	0.854		5.978	
F2 treated mucin RT 5 min-1	0.554	1.285	10.025	0.431		7.802	
F2 treated mucin RT 5 min-2	1.011	1.189	9.771	0.850	0.685	8.218	7.220
F2 treated mucin RT 5 min-3	1.386	1.789	10.091	0.775		5.641	

* Each spectrum was normalized and integrated by OPUS 7.2 (Bruker, Germany).

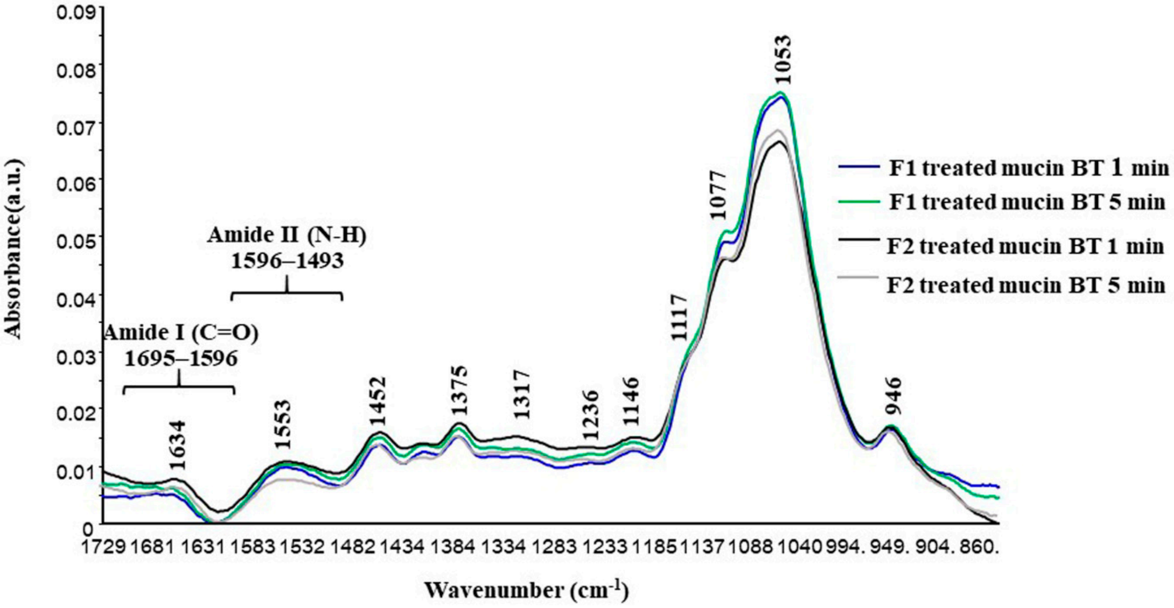

Figure S2. ATR-FTIR spectra of F1- and F2-treated mucin at BT for 1 min and 5 min, 1634 cm^{-1} (C=O stretching of amide I), 1553 cm^{-1} (C–N stretching of amide II), 1452 cm^{-1} (C–H bending), 1375 cm^{-1} (CH_3 bending), 1317 cm^{-1} and 1236 cm^{-1} (amide III), 1146 and 1117 cm^{-1} (C–O stretching), 1077 cm^{-1} (C–N stretching), 1053 cm^{-1} (C–O stretching or C-O bending of carbohydrates), and 946 cm^{-1} (C–H out-of-plane bending). Each spectrum was averaged from 3 spectra.

Table S2. The absorption intensities under the spectra of amide I, amide II and carbohydrate, ratio of amide I/amide II and ratio of carbohydrate/amide II from F1- and F2-treated mucin at BT for 1 min and 5 min.

Peak area (raw data)	Peak1	Peak2	Peak3	Ratio	Average	Ratio	Average
Samples/Wave number(cm-1)	Amide I	Amide II	Carbo	AmideI/amideII	AmideI/amideII	Carbo/AmideII	Carbo/AmideII
	1695–1596.2	1596.2–1492.6	1189.1–972.7				
F1 treated mucin BT 1 min-1	-0.175	0.781	11.801	-0.224		15.110	
F1 treated mucin BT 1 min-2	0.269	0.653	11.797	0.412	-0.062	18.066	20.879
F1 treated mucin BT 1 min-3	-0.164	0.44	12.963	-0.373		29.461	
F1 treated mucin BT 5 min-1	-0.076	0.472	12.911	-0.161		27.354	
F1 treated mucin BT 5 min-2	0.159	0.457	12.322	0.348	-0.086	26.963	23.009
F1 treated mucin BT 5 min-3	-0.312	0.702	10.327	-0.444		14.711	
F2 treated mucin BT 1 min-1	-0.239	0.369	9.377	-0.648		25.412	
F2 treated mucin BT 1 min-2	0.144	0.701	10.614	0.205	0.225	15.141	18.870
F2 treated mucin BT 1 min-3	0.815	0.729	11.705	1.118		16.056	
F2 treated mucin BT 5 min-1	-0.021	0.798	7.971	-0.026		9.989	
F2 treated mucin BT 5 min-2	0.455	0.363	11.744	1.253	0.395	32.353	32.227
F2 treated mucin BT 5 min-3	-0.009	0.22	11.955	-0.041		54.341	

* Each spectrum was normalized and integrated by OPUS 7.2 (Bruker, Germany).

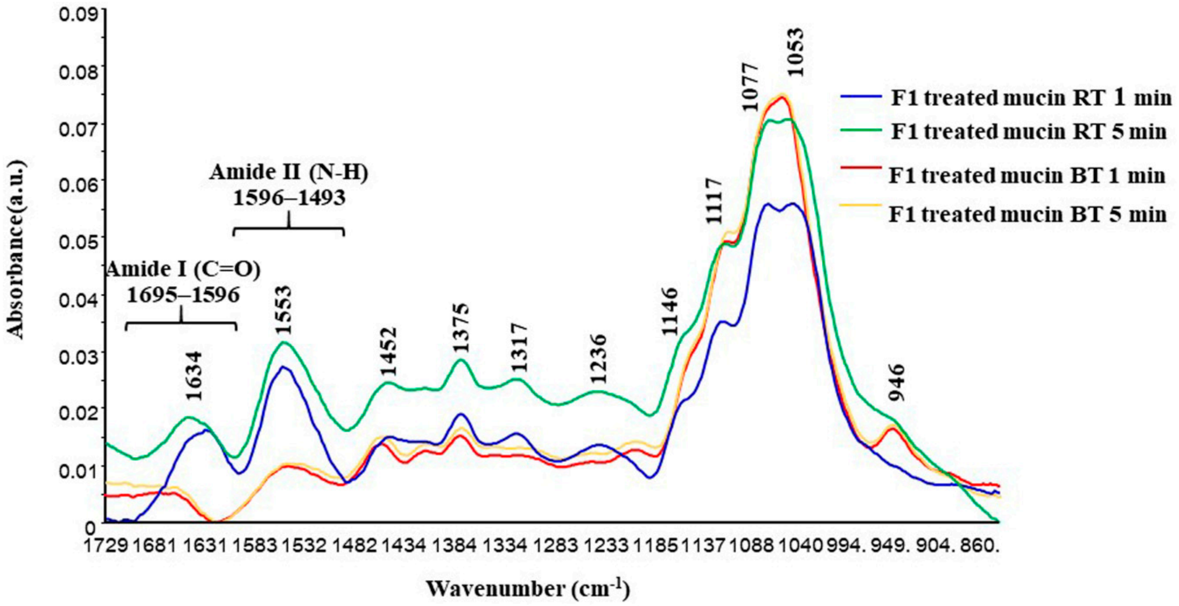

Figure S3. ATR-FTIR spectra of F1 treated mucin at RT and BT for 1 min and 5 min, 1634 cm^{-1} (C=O stretching of amide I), 1553 cm^{-1} (C–N stretching of amide II), 1452 cm^{-1} (C–H bending), 1375 cm^{-1} (CH_3 bending), 1317 cm^{-1} and 1236 cm^{-1} (amide III), 1146 and 1117 cm^{-1} (C–O stretching), 1077 cm^{-1} (C–N stretching), 1053 cm^{-1} (C–O stretching or C–O bending of carbohydrates), and 946 cm^{-1} (C–H out-of-plane bending). Each spectrum was averaged from 3 spectra.

Table S3. The absorption intensities under the spectra of amide I, amide II and carbohydrate, ratio of amide I/amide II and ratio of carbohydrate/amide II from F1 treated mucin at RT and BT for 1 min and 5 min.

Peak area (raw data) Samples/Wave number(cm-1)	Peak1 Amide I 1695–1596.2	Peak2 Amide II 1596.2–1492.6	Peak3 Carbo 1189.1–972.7	Ratio AmideI/amideII	Average AmideI/amideII	Ratio Carbo/AmideII	Average Carbo/AmideII
F1 treated mucin RT 1 min-1	1.240	1.822	9.214	0.681		5.057	
F1 treated mucin RT 1 min-2	1.129	1.832	8.951	0.616	0.588	4.886	5.024
F1 treated mucin RT 1 min-3	0.961	2.062	10.578	0.466		5.130	
F1 treated mucin RT 5 min-1	0.804	1.653	10.093	0.486		6.106	
F1 treated mucin RT 5 min-2	0.783	1.858	9.816	0.421	0.349	5.283	5.838
F1 treated mucin RT 5 min-3	0.181	1.309	8.019	0.138		6.126	
F2 treated mucin BT 1 min-1	-0.239	0.369	9.377	-0.648		25.412	
F2 treated mucin BT 1 min-2	0.144	0.701	10.614	0.205	0.225	15.141	18.870
F2 treated mucin BT 1 min-3	0.815	0.729	11.705	1.118		16.056	
F2 treated mucin BT 5 min-1	-0.021	0.798	7.971	-0.026		9.989	
F2 treated mucin BT 5 min-2	0.455	0.363	11.744	1.253	0.395	32.353	32.227
F2 treated mucin BT 5 min-3	-0.009	0.22	11.955	-0.041		54.341	

* Each spectrum was normalized and integrated by OPUS 7.2 (Bruker, Germany).

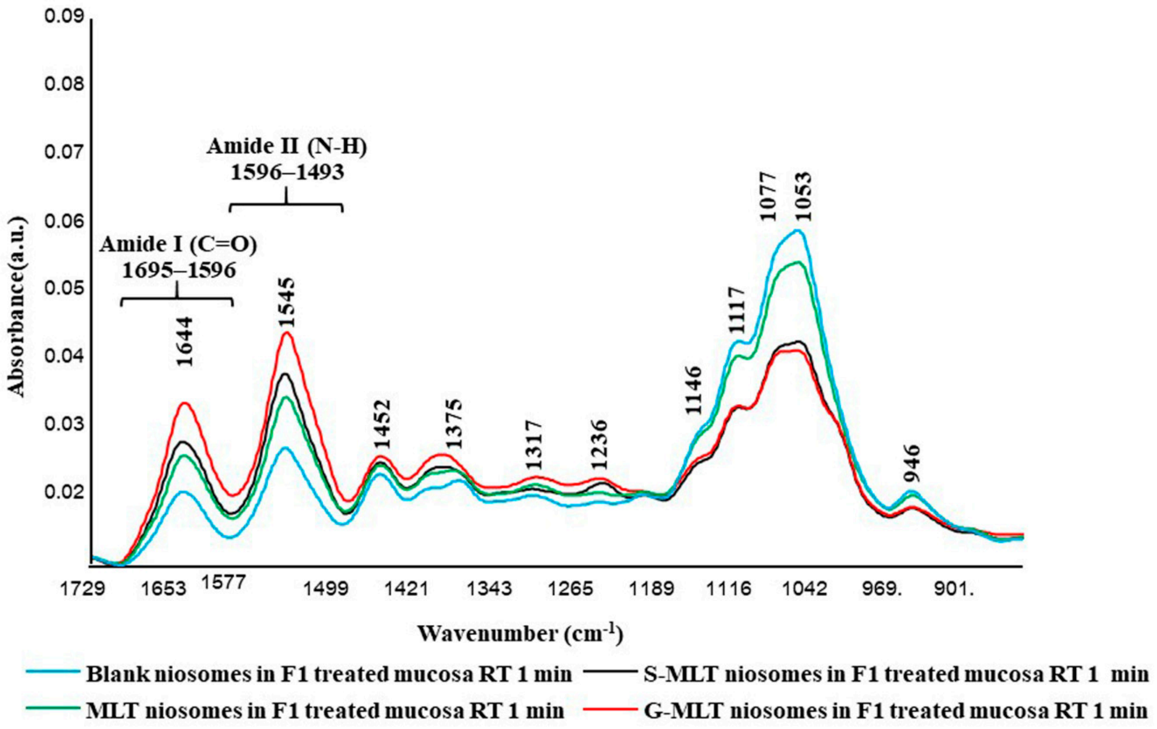

Figure S4. ATR-FTIR spectra of blank, melatonin and its derivative niosomes in F1 treated mucosa at RT for 1 min, 1644 cm^{-1} (C=O stretching of amide I), 1545 cm^{-1} (N–H bending and C–N stretching of amide II), 1452 cm^{-1} and 1397 cm^{-1} (C–H bending), 1236 cm^{-1} (amide III), 1146 cm^{-1} and 1117 cm^{-1} (C–O stretching), 1077 cm^{-1} (C-N stretching), and 1053 cm^{-1} (C–O stretching or C–O bending of carbohydrates), and 946 cm^{-1} (C–H out-of-plane bending). Each spectrum was averaged from 3 spectra.

Table S4. The absorption intensities under the spectra of amide I, amide II and carbohydrate, ratio of amide I/amide II and ratio of carbohydrate/amide II from blank, melatonin and its derivative niosomes in F1 treated mucosa at RT for 1 min.

Peak area (raw data)	Peak1	Peak2	Peak3	Ratio	Average	Ratio	Average
Samples/Wave number(cm-1)	Amide I	Amide II	Carbo	AmideI/amideII	AmideI/amideII	Carbo/AmideII	Carbo/AmideII
	1695–1596.2	1596.2–1492.6	1189.1–972.7				
BNG treated mucosa RT 1 min-1	0.374	0.823	11.23	0.454		13.645	
BNG treated mucosa RT 1 min-2	0.744	1.563	9.959	0.476	0.553	6.372	7.613
BNG treated mucosa RT 1 min-3	2.017	2.765	7.802	0.729		2.822	
MNG treated mucosa RT 1 min-1	1.134	1.605	8.739	0.707		5.445	
MNG treated mucosa RT 1 min-2	1.076	1.482	8.957	0.726	0.716	6.044	4.722
MNG treated mucosa RT 1 min-3	1.731	2.415	6.465	0.717		2.677	
SNG treated mucosa RT 1 min-1	1.301	1.811	5.839	0.718		3.224	
SNG treated mucosa RT 1 min-2	2.398	2.828	3.01	0.848	0.769	1.064	2.289
SNG treated mucosa RT 1 min-3	1.588	2.144	5.526	0.741		2.577	
GNG treated mucosa RT 1 min-1	1.563	2.245	5.778	0.696		2.574	
GNG treated mucosa RT 1 min-2	2.084	2.684	5.649	0.776	0.743	2.105	2.202
GNG treated mucosa RT 1 min-3	2.081	2.753	5.307	0.756		1.928	

BNG= blank niosomes in F1 treated mucosa, MNG = MLT niosomes in F1, SNG = S-MLT niosomes in F1, GNG= G-MLT niosomes in F1

* Each spectrum was normalized and integrated by OPUS 7.2 (Bruker, Germany)

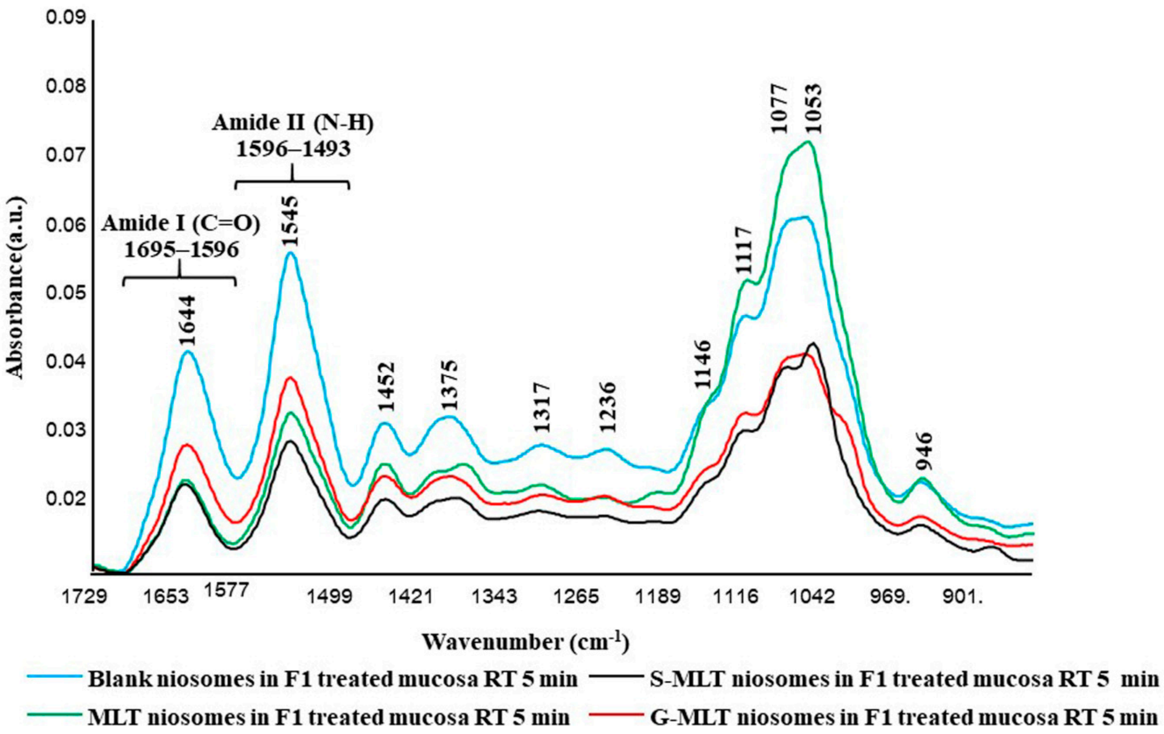

Figure S5. ATR-FTIR spectra of blank, melatonin and its derivative niosomes in F1 treated mucosa at RT for 5 min, 1644 cm^{-1} (C=O stretching of amide I), 1545 cm^{-1} (N–H bending and C–N stretching of amide II), 1452 cm^{-1} and 1397 cm^{-1} (C–H bending), 1236 cm^{-1} (amide III), 1146 cm^{-1} and 1117 cm^{-1} (C–O stretching), 1077 cm^{-1} (C–N stretching), and 1053 cm^{-1} (C–O stretching or C–O bending of carbohydrates), and 946 cm^{-1} (C–H out-of-plane bending). Each spectrum was averaged from 3 spectra.

Table S5. The absorption intensities under the spectra of amide I, amide II and carbohydrate, ratio of amide I/amide II and ratio of carbohydrate/amide II from blank, melatonin and its derivative niosomes in F1 treated mucosa at RT for 5 min.

Peak area (raw data)	Peak1	Peak2	Peak3	Ratio	Average	Ratio	Average
Samples/Wave number(cm-1)	Amide I 1695–1596.2	Amide II 1596.2–1492.6	Carbo 1189.1–972.7	AmideI/amideII	AmideI/amideII	Carbo/AmideII	Carbo/AmideII
BNG treated mucosa RT 5 min-1	1.653	2.348	8.589	0.704		3.658	
BNG treated mucosa RT 5 min-2	2.266	2.981	7.437	0.760	0.754	2.495	2.760
BNG treated mucosa RT 5 min-3	2.514	3.155	6.716	0.797		2.129	
MNG treated mucosa RT 5 min-1	1.349	1.729	7.236	0.780		4.185	
MNG treated mucosa RT 5 min-2	0.87	1.281	9.161	0.679	0.701	7.151	6.918
MNG treated mucosa RT 5 min-3	0.668	1.039	9.784	0.643		9.417	
SNG treated mucosa RT 5 min-1	1.096	1.606	6.632	0.682		4.130	
SNG treated mucosa RT 5 min-2	1.069	1.529	6.991	0.699	0.710	4.572	3.724
SNG treated mucosa RT 5 min-3	1.612	2.15	5.308	0.750		2.469	
GNG treated mucosa RT 5 min-1	1.13	1.657	6.215	0.682		3.751	
GNG treated mucosa RT 5 min-2	2.074	2.864	4.816	0.724	0.709	1.682	2.848
GNG treated mucosa RT 5 min-3	1.501	2.079	6.469	0.722		3.112	

BNG= blank niosomes in F1 treated mucosa, MNG = MLT niosomes in F1, SNG = S-MLT niosomes in F1, GNG= G-MLT niosomes in F1.

* Each spectrum was normalized and integrated by OPUS 7.2 (Bruker, Germany).

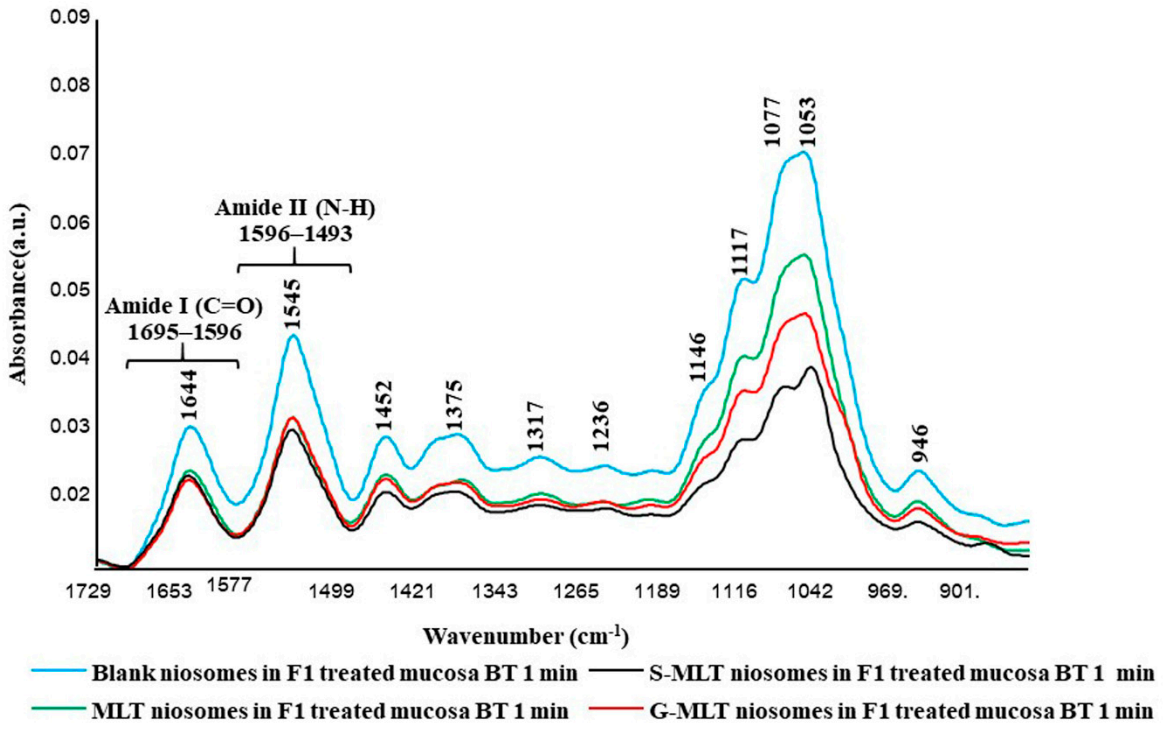

Figure S6. ATR-FTIR spectra of blank, melatonin and its derivative niosomes in F1 treated mucosa at BT for 1 min, 1644 cm^{-1} (C=O stretching of amide I), 1545 cm^{-1} (N–H bending and C–N stretching of amide II), 1452 cm^{-1} and 1397 cm^{-1} (C–H bending), 1236 cm^{-1} (amide III), 1146 cm^{-1} and 1117 cm^{-1} (C–O stretching), 1077 cm^{-1} (C–N stretching), and 1053 cm^{-1} (C–O stretching or C–O bending of carbohydrates), and 946 cm^{-1} (C–H out-of-plane bending). Each spectrum was averaged from 3 spectra.

Table S6. The absorption intensities under the spectra of amide I, amide II and carbohydrate, ratio of amide I/amide II and ratio of carbohydrate/amide II from blank, melatonin and its derivative niosomes in F1 treated mucosa at BT for 1 min.

Peak area (raw data) Samples/Wave number(cm-1)	Peak1 Amide I 1695–1596.2	Peak2 Amide II 1596.2–1492.6	Peak3 Carbo 1189.1–972.7	Ratio AmideI/amideII	Average AmideI/amideII	Ratio Carbo/AmideII	Average Carbo/AmideII
BNG treated mucosa BT 1 min-1	0.632	1.328	10.335	0.476		7.782	
BNG treated mucosa BT 1 min-2	1.865	2.698	7.911	0.691	0.607	2.932	4.853
BNG treated mucosa BT 1 min-3	1.509	2.311	8.886	0.653		3.845	
MNG treated mucosa BT 1 min-1	1.272	1.747	8.431	0.728		4.826	
MNG treated mucosa BT 1 min-2	0.997	1.518	9.047	0.657	0.710	5.960	5.003
MNG treated mucosa BT 1 min-3	1.449	1.943	8.208	0.746		4.224	
SNG treated mucosa BT 1 min-1	1.102	1.657	6.317	0.665		3.812	
SNG treated mucosa BT 1 min-2	1.724	2.322	3.704	0.742	0.694	1.595	3.112
SNG treated mucosa BT 1 min-3	1.093	1.618	6.357	0.676		3.929	
GNG treated mucosa BT 1 min-1	1.106	1.763	7.14	0.627		4.050	
GNG treated mucosa BT 1 min-2	1.354	2.086	6.336	0.649	0.603	3.037	4.002
GNG treated mucosa BT 1 min-3	0.837	1.569	7.719	0.533		4.920	

BNG= blank niosomes in F1 treated mucosa, MNG = MLT niosomes in F1, SNG = S-MLT niosomes in F1, GNG= G-MLT niosomes in F1.

* Each spectrum was normalized and integrated by OPUS 7.2 (Bruker, Germany)

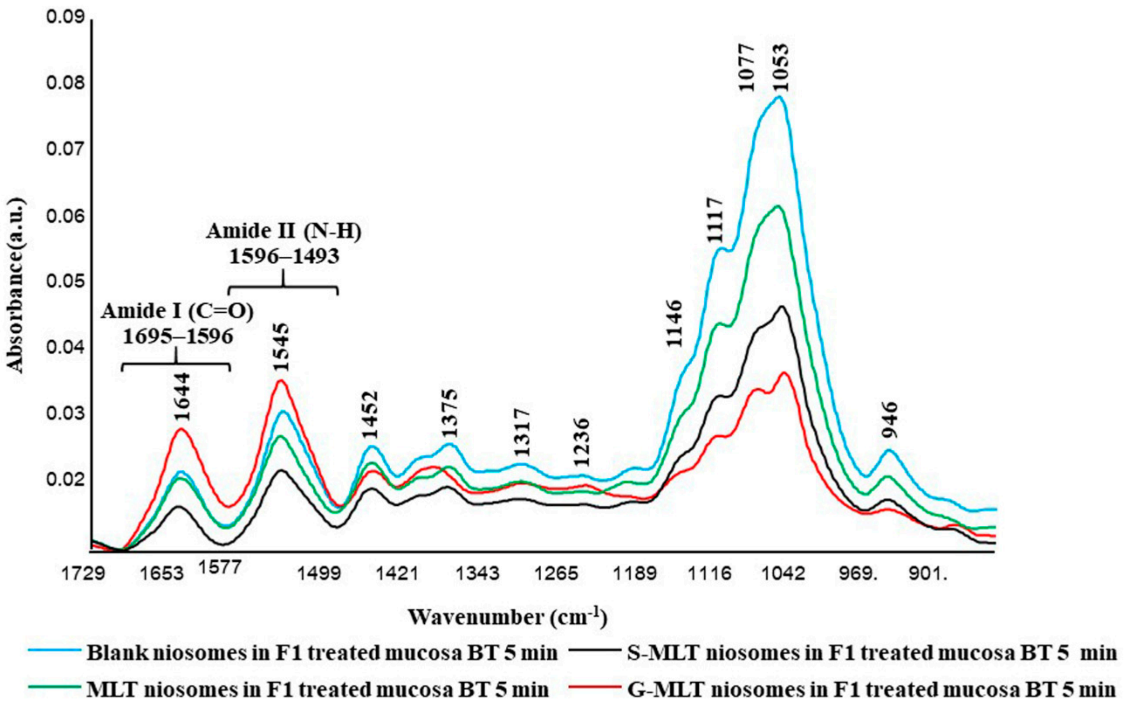

Figure S7. ATR-FTIR spectra of blank, melatonin and its derivative niosomes in F1 treated mucosa at BT for 5 min, 1644 cm^{-1} (C=O stretching of amide I), 1545 cm^{-1} (N-H bending and C–N stretching of amide II), 1452 cm^{-1} and 1397 cm^{-1} (C–H bending), 1236 cm^{-1} (amide III), 1146 cm^{-1} and 1117 cm^{-1} (C–O stretching), 1077 cm^{-1} (C–N stretching), and 1053 cm^{-1} (C–O stretching or C–O bending of carbohydrates), and 946 cm^{-1} (C–H out-of-plane bending). Each spectrum was averaged from 3 spectra.

Table S7. The absorption intensities under the spectra of amide I, amide II and carbohydrate, ratio of amide I/amide II and ratio of carbohydrate/amide II from blank, melatonin and its derivative niosomes in F1 treated mucosa at BT for 5 min.

Peak area (raw data) Samples/Wave number(cm-1)	Peak1 Amide I 1695–1596.2	Peak2 Amide II 1596.2–1492.6	Peak3 Carbo 1189.1–972.7	Ratio AmideI/amideII	Average AmideI/amideII	Ratio Carbo/AmideII	Average Carbo/AmideII
BNG treated mucosa BT 5 min-1	1.501	2.216	8.817	0.677		3.979	
BNG treated mucosa BT 5 min-2	0.761	1.319	10.874	0.577	0.552	8.244	8.411
BNG treated mucosa BT 5 min-3	0.345	0.858	11.163	0.402		13.010	
MNG treated mucosa BT 5 min-1	2.001	2.415	6.729	0.829		2.786	
MNG treated mucosa BT 5 min-2	0.449	0.84	10.047	0.535	0.659	11.961	8.325
MNG treated mucosa BT 5 min-3	0.604	0.983	10.054	0.614		10.228	
SNG treated mucosa BT 5 min-1	0.263	0.911	7.87	0.289		8.639	
SNG treated mucosa BT 5 min-2	1.459	1.944	5.881	0.751	0.513	3.025	6.636
SNG treated mucosa BT 5 min-3	0.471	0.94	7.75	0.501		8.245	
GNG treated mucosa BT 5 min-1	1.403	2.034	6.189	0.690		3.043	
GNG treated mucosa BT 5 min-2	1.78	2.57	4.834	0.693	0.701	1.881	2.678
GNG treated mucosa BT 5 min-3	1.501	2.079	6.469	0.722		3.112	

BNG= blank niosomes in F1 treated mucosa, MNG = MLT niosomes in F1, SNG = S-MLT niosomes in F1, GNG= G-MLT niosomes in F1.

* Each spectrum was normalized and integrated by OPUS 7.2 (Bruker, Germany)

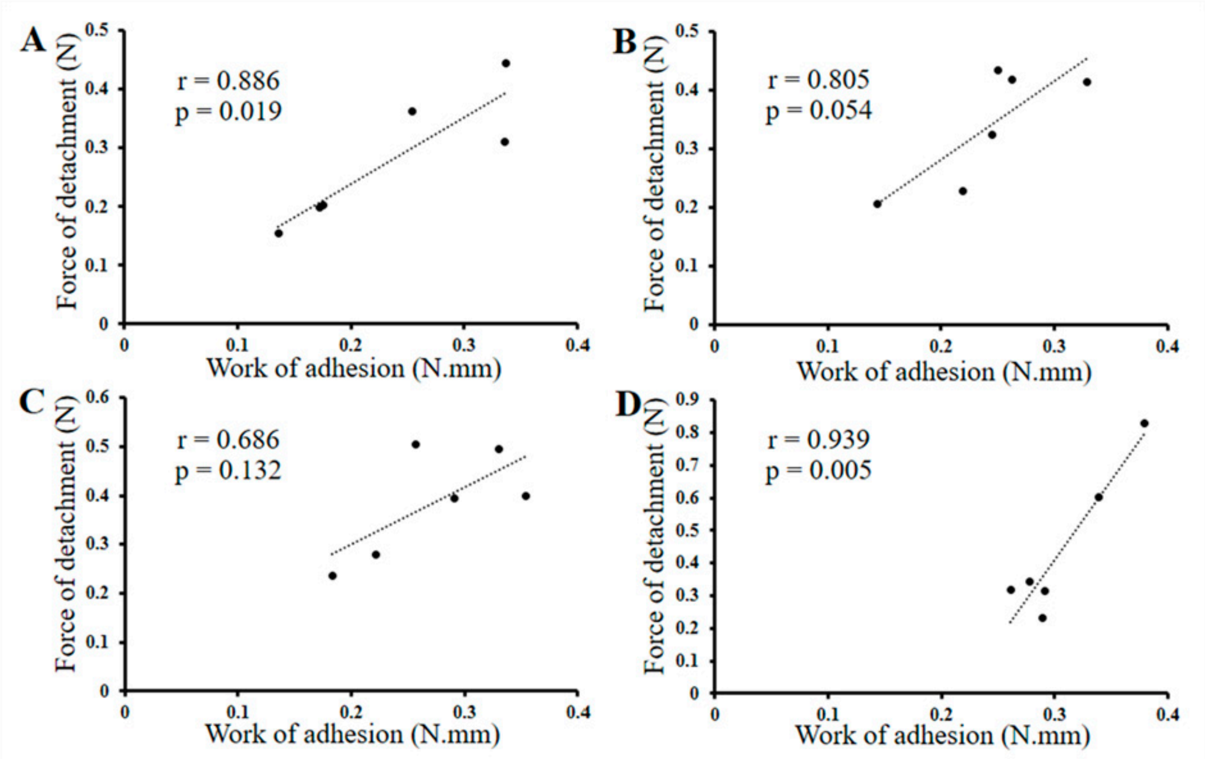

Figure S8. The correlation (r) of force of detachment and work of adhesion between gels (Blank niosomes in F1 (**A**)**,** MLT niosomes in F1 (**B**)**, S-**MLT niosomes in F1 (**C**), and G-MLT niosomes in F1 (**D**) and mucosa, * p is *p*-value.

Table S8. The absorption intensities under the spectra of amide I, amide II and carbohydrate, ratio of amide I/amide II and ratio of carbohydrate/amide II from F1 treated mucin/untreated mucin ratio at BT and RT for 1 min and 5 min.

Peak area (raw data)	Peak1	Peak2	Peak3	Ratio	Ratio	ratio	Average	ratio	Average
Samples/Wave number(cm-1)	Amide I	Amide II	Carbo	AmideI/amideII (i)	Carbo/ amideII (ii)	i/mucin-a	i/mucin-a	ii/mucin-c	ii/mucin-c
	1695–1596.2	1596.2–1492.6	1189.1–972.7						
F1 treated mucin BT 1 min-1	-0.175	0.781	11.801	-0.224	15.110	-0.248		3.462	
F1 treated mucin BT 1 min-2	0.269	0.653	11.797	0.412	18.066	0.456	-0.068	4.140	4.784
F1 treated mucin BT 1 min-3	-0.164	0.44	12.963	-0.373	29.461	-0.412		6.751	
F1 treated mucin BT 5 min-1	-0.076	0.472	12.911	-0.161	27.354	-0.178		6.268	
F1 treated mucin BT 5 min-2	0.159	0.457	12.322	0.348	26.963	0.385	-0.095	6.178	5.272
F1 treated mucin BT 5 min-3	-0.312	0.702	10.327	-0.444	14.711	-0.492		3.371	
F1 treated mucin RT 1 min-1	1.24	1.822	9.214	0.681	5.057	0.753		1.159	
F1 treated mucin RT 1 min-2	1.129	1.832	8.951	0.616	4.886	0.682	0.650	1.120	1.151
F1 treated mucin RT 1 min-3	0.961	2.062	10.578	0.466	5.130	0.516		1.175	
F1 treated mucin RT 5 min-1	0.804	1.653	10.093	0.486	6.106	0.538		1.399	
F1 treated mucin RT 5 min-2	0.783	1.858	9.816	0.421	5.283	0.466	0.386	1.211	1.338
F1 treated mucin RT 5 min-3	0.181	1.309	8.019	0.138	6.126	0.153		1.404	

mucin-a =average amide I/amide II of mucin = 0.904
mucin-c = average carbohydrate/amide II of mucin = 4.364
i = ratio of amide I/amide II, ii = ratio of carbohydrate/amide II
* Each spectrum was normalized and integrated by OPUS 7.2 (Bruker, Germany)

Table S9. The absorption intensities under the spectra of amide I, amide II and carbohydrate, ratio of amide I/amide II and ratio of carbohydrate/amide II from F1, F2, blank niosomes (BNG), MLT niosomes (MNG), S-MLT niosomes (SNG), G-MLT niosomes (GNG), mucin and mucosa.

Peak area (raw data)	Peak1	Peak2	Peak3	Ratio	Average	Ratio	Average
Samples/Wave number(cm-1)	Amide I	Amide II	Carbo	AmideI/amideII	AmideI/amideII	Carbo/AmideII	Carbo/AmideII
	1695–1596.2	1596.2–1492.6	1189.1–972.7				
F1-1	-0.231	0.276	12.498	-0.837		45.283	
F1-2	-0.371	0.298	11.932	-1.245	-0.868	40.040	46.372
F1-3	-0.126	0.242	13.018	-0.521		53.793	
F2-1	0.149	0.098	12.144	1.520		123.918	
F2-2	0.121	0.080	12.437	1.513	1.530	155.463	140.373
F2-3	0.137	0.088	12.473	1.557		141.739	
BNG-1	-0.217	0.268	11.389	-0.810		42.496	
BNG-2	-0.170	0.292	11.963	-0.582	-0.661	40.969	44.488
BNG-3	-0.142	0.240	12.000	-0.592		50.000	
MNG-1	-0.083	0.160	11.356	-0.519		70.975	
MNG-2	-0.029	0.142	11.381	-0.204	-0.403	80.148	77.260
MNG-3	-0.068	0.140	11.292	-0.486		80.657	
SNG-1	-0.171	0.209	9.643	-0.818		46.139	
SNG-2	-0.153	0.180	10.141	-0.850	-0.825	56.339	50.809
SNG-3	-0.160	0.198	9.890	-0.808		49.949	
SNG-1	-0.171	0.209	9.643	-0.818		46.139	
SNG-2	-0.153	0.180	10.141	-0.850	-0.825	56.339	50.809
SNG-3	-0.160	0.198	9.890	-0.808		49.949	
Mucin-1	1.654	1.896	7.856	0.872		4.143	
Mucin-2	1.609	1.877	8.273	0.857	0.867	4.408	4.361
Mucin-3	1.579	1.809	8.196	0.873		4.531	
Mucosa3-1	3.699	3.989	1.359	0.927		0.341	
Mucosa3-2	3.608	4.051	1.345	0.891	0.906	0.332	0.352
Mucosa3-3	3.729	4.141	1.588	0.901		0.383	

BNG= blank niosomes, MNG = MLT niosomes, SNG = S-MLT niosomes, GNG= G-MLT niosomes.

* Each spectrum was normalized and integrated by OPUS 7.2 (Bruker, Germany)
